# *Not just reproductive*: a roundtable on addressing gynaecological health through the life course in South Asia

**DOI:** 10.1080/26410397.2025.2521031

**Published:** 2025-06-18

**Authors:** Sapna Desai, Kaosar Afsana, Neymat Chadha, Dipti Govil, Neha Mankani, Ramya Kumar

**Affiliations:** a Senior Fellow, Population Council Institute, SAHELI Consortium, New Delhi, India. *Correspondence*: sapna.i.desai@gmail.com; b Professor, BRAC JPG School of Public Health, Dhaka, Bangladesh; c Research Fellow, The George Institute for Global Health India, SAHELI Consortium, New Delhi, India; d Assistant Professor, International Institute for Population Sciences, SAHELI Consortium, Mumbai; e Founder, Mama Baby Fund, Karachi, Pakistan; f Senior Lecturer, Department of Community and Family Medicine, University of Jaffna, Jaffna, Sri Lanka

**Keywords:** women’s health, gynaecology, South Asia, sexual and reproductive health, life course

## Abstract

A roundtable on gynaecological health in South Asia engaged speakers from four countries to share insights on the current context, challenges and priorities for action in the region. Women’s gynaecological needs beyond maternal health are overlooked in most settings, with common ailments underdiagnosed or untreated. While over-medicalisation and a lack of data and evidence are two common challenges, midwife-led programmes, investment in primary health care and nationwide data collection on gynaecological health are promising initiatives. The key priorities for evidence generation are to: understand gynaecological health and its interlinked determinants; examine impacts on quality of life; and design approaches that address women's health through the life course.

## Résumé

Une table ronde sur la santé gynécologique en Asie du Sud a rassemblé des intervenants de quatre pays qui ont partagé leurs points de vue sur le contexte actuel, les défis et les priorités d’action dans la région. Les besoins gynécologiques des femmes, au-delà de la santé maternelle, ne sont pas pris en compte dans la plupart des pays, certaines affections courantes étant sous-diagnostiquées ou non traitées. Si la surmédicalisation et le manque de données probantes constituent deux défis communs, les programmes dirigés par des sages-femmes, les investissements dans les soins de santé primaires et le recueil de données nationales sur la santé gynécologique représentent des initiatives prometteuses. La priorité pour la production future de données est de comprendre la santé gynécologique, ses déterminants interdépendants et ses conséquences sur la qualité de vie, et de concevoir des approches adaptées à la santé des femmes tout au long de leur vie.

## Resumen

En una mesa redonda sobre la salud ginecológica en Asia meridional, oradores de cuatro países compartieron sus perspectivas sobre el contexto actual, los retos y las prioridades de acción en la región. En la mayoría de los contextos, se pasan por alto las necesidades ginecológicas de las mujeres más allá de la salud materna, y las dolencias comunes son subdiagnosticadas o no tratadas. Si bien la sobremedicalización y la falta de datos y evidencia son dos retos comunes, los programas liderados por parteras, las inversiones en servicios de atención primaria y la recolección de datos sobre salud ginecológica a nivel nacional son iniciativas prometedoras. La prioridad clave para generar evidencia futura es entender la salud ginecológica, sus determinantes interconectados y su impacto en la calidad de vida, y formular estrategias que aborden la salud de las mujeres a lo largo del ciclo de vida.

## Introduction

Across South Asia, women’s health is largely equated with reproductive and maternal health, with limited services that address sex-specific or gender-related needs across the life course.^1^ As a result, women’s engagement with health systems is mediated by reproduction: starting with menstrual care for adolescents, progressing to antenatal care for pregnant women and culminating in delivery and postnatal care. Sex-specific conditions not directly linked to pregnancy, such as menstrual disorders or cervical cancer, are not high priority investments in most countries. Yet living with gynaecological conditions such as heavy menstrual bleeding or endometriosis has considerable impacts on women’s quality of life.^2,3^ A consistent, and growing, body of evidence indicates that reproductive life events, such as age at menarche, history of menstrual disorders, hysterectomy and early menopause, are linked to later risk of non-communicable diseases such as diabetes, cardiovascular disease, osteoporosis and mental health.^4–7^ Further, climate change (heat, cyclone/floods, environmental degradation, reduced agricultural production) has complex connections to women’s sexual and reproductive health including risks of violence against women.^8^

Accordingly, discourse and actions on women’s health and sexual and reproductive health and rights (SRHR) in South Asia must expand beyond reproduction. As a start, we held a roundtable to examine the status of women’s gynaecological health in the region and its sociocultural, economic and health system determinants. Through a conversation with practitioners and researchers in India, Bangladesh, Sri Lanka and Pakistan, we aimed to identify shared concerns and opportunities to learn from experience, ultimately towards a common action agenda. The roundtable was convened by the Study and Action on Hysterectomy: Evidence on women’s health through the Life course in India (SAHELI), a consortium based in India that examines hysterectomy and gynaecological health^9^; Sexual and Reproductive Health Matters South Asia Hub, which serves as a knowledge platform for the region^10^; and the Health Equity Network (HENI), a network of Indian researchers working on evidence and action on health inequities.

Roundtable speakers included: Dipti Govil, a researcher on hysterectomy, menopause and ageing in India; Kaosar Afsana, a researcher and programme implementer on reproductive health in Bangladesh; Ramya Kumar, a medical doctor and researcher in Sri Lanka; and Neha Mankani, a practicing midwife and advocate in Pakistan. The discussion was moderated by SAHELI researchers Sapna Desai and Neymat Chadha with participation by approximately 50 researchers, practitioners and students across South Asia. The 90-minute discussion was conducted via Zoom in December 2024, structured around three questions:

1. What are the major issues facing women's health in your country, and within those challenges, how does gynaecological health feature as a priority?
2. What are the main initiatives your country is implementing to address gynaecological health?
3. What are the key priorities for policy advocacy and organising moving forward?

The webinar was recorded and transcribed, after which we identified key themes. The author group reflected on findings and organised this article by key issues and priorities for action. All quotes are included with permission of the speakers, who are also authors of the article.

## Country settings

The four settings discussed, while only some of the eight countries of South Asia, provided a diverse mix of health systems and trajectories in women’s health. All countries have seen improvements in maternal health outcomes over the past two decades. Fertility and access to contraception have stabilised, though relatively slower in Pakistan. Expenditure on health remains low in most settings, contributing to privatisation across the region and limited access to a continuum of care for women in the public sector. Sri Lanka stands out with higher current health expenditure as a percent of GDP, and a free healthcare system at the point of use, which along with free education, have contributed to impressive maternal health outcomes relative to other countries in the region.^11^ Abortion is criminalised in most countries, with the exception of India and Nepal. As countries shift away from maternal health-centric outcomes to achieving an expanded primary health care approach, each faces new challenges related to changing burden of disease, urbanisation and ageing – as well as persistent inequities in access to services (Table 1).^12^

**Table 1.** Overview of Bangladesh, India, Pakistan and Sri Lanka

Indicator	Bangladesh	India	Pakistan	Sri Lanka
Total population, 2023^1^	171.5 mil	1.4 bill	247.5 mil	22.0 mil
Total fertility rate, 2023^1^	2.2	2.0	3.6	2.0
Adult female literacy rate^2^	74	70	46	92
GDP per capita 2023 (US$)^3^	2551	2481	1365	3828
CHE* as % of GDP (2022)^4^	2.4	3.3	2.9	4.4
Skilled birth attendance (% births)^5^	59	89	68	100
Maternal mortality ratio, 2023^6^	115	80	155	18

*Current Health Expenditure.

Source*: All data accessed from World Bank Open Data (23 April 2025).* ^1^United Nations Population Division. World Population Prospects: 2024 Revised Estimates. ^2^UNESCO Institute for Statistics, years: Bangladesh, 2021; India, 2023; Pakistan, 2019; Sri Lanka, 2022. ^3^World Bank National Accounts Data. ^4^World Health Organization Global Health Expenditure database. ^5^Demographic Health Surveys years: Bangladesh, 2019; India, 2021; Pakistan, 2021; Sri Lanka, 2016; ^6^WHO, UNICEF, UNFPA, World Bank Group, and UNDESA/Population Division. Trends in maternal mortality estimates 2000 to 2023. Geneva, World Health Organization, 2025.

## Gynaecological health: context within women’s health

We first explored the framing of women’s health and specifically policies and programmes for gynaecological health in the four countries. In Bangladesh, menstrual health, contraception and pregnancy are the starting point for women’s health, alongside a steady expansion in services after the 1994 International Conference on Population and Development.^13^ Gynaecological issues such as dysmenorrhea, heavy bleeding and uterine problems have a large impact on women’s lives, productivity and well-being in both rural and urban contexts. However, few services are designed to address issues beyond maternal health. Reproductive tract infections, while common and preventable, are not addressed adequately in public health initiatives. While the government has recently initiated HPV vaccination for women and girls to address a rise in cervical cancer, broader prevention measures are required. A general lack of awareness and advocacy campaigns for women’s health hinder the implementation of simple and effective preventive measures like regular cervical visual inspection with ascetic acid.

Like Bangladesh, India also grapples with a range of women's health issues that go beyond reproduction. Common gynaecological concerns include reproductive tract infections, menstrual irregularities, dysmenorrhea, endometriosis, fibrosis and polycystic ovarian syndrome (PCOS). Further, women’s health/illness also encompasses non-reproductive elements such as violence, nutrition, cardiovascular diseases, bone health, mental health and urinary tract disorders. India’s National Health Mission covers a broad ambit of services, with women-specific initiatives such as the Janani Suraksha Yojana (JSY) for institutional delivery and cancer screening. Overall, however, programmes for women mainly address maternal health, while the specific healthcare needs of women beyond childbearing age are often overlooked and inadequately addressed. As a roundtable participant observed:

*“Once women transition from reproductive to non-reproductive stages – either through cessation of menstruation or sterilisation – their health needs usually fall outside the scope of the health delivery system. Instead, health policies focus on reproductive health and old age, neglecting the health needs of middle-aged women.”*

Sri Lanka's health system provides a range of women's health services, primarily through the public sector. The preventive health system is well organised: public health midwives register eligible families, focusing on maternal and child health, and conduct home visits and polyclinics. The country initiated Well Woman clinics for women over age 35 to access cervical cancer screening, clinical breast exams and other services in the 1990s.^14^ While it has expanded since, the system is strained by health worker shortages, primarily affecting rural and war-affected areas. Women use both outpatient departments of public hospitals or private sector providers for gynaecological issues. Menopause and perimenopause treatments are highly medicalised and primarily available in the private sector, with limited emphasis on lifestyle modification. Breast cancer rates are rising, but screening mammography is not freely available through the public system, and less-invasive breast conserving surgery is difficult to access. Despite these challenges, the infrastructure exists for women to access community-based services and referrals to secondary and tertiary care centres.

In Pakistan, the demand for women's health services far exceeds available resources, particularly in the public sector. While a significant number of midwives provide obstetric care, quality issues persist, leading many women to rely on inconsistent private health care, including small, unregulated private clinics that often provide low-quality care. Within this landscape, midwives play a critical role in service delivery. In a promising example of a holistic approach to women's health – one that considers the connections between reproduction and health through the life course – Mama Baby Fund, a not-for-profit organisation in Pakistan, commenced providing midwife-led care in an inaccessible island community through a free pregnancy and well-baby clinic. The clinic expanded to address broader gynaecological health and women's wellness.^15^ For instance, complications during childbirth, such as prolonged labour and poorly managed tears, can lead to long-term problems like pelvic floor damage – resulting in urinary/faecal incontinence, infections and chronic pain – that significantly impact women’s quality of life. High c-section rates in Pakistan contribute to dangerous complications like placenta accreta, which in turn often result in hysterectomies at a young age. Psychological trauma from birth experiences also impacts women's health through the life course, while disrespect and abuse during childbirth can impact a woman's overall well-being, including her sexual health. Although midwives working in the non-profit sector provide some of these services and safe care, there are limitations to what they can provide in the absence of governmental support for gynaecological health.

Across settings, panellists noted that deeply ingrained social and cultural norms influence women's health, with a paternalistic approach from healthcare providers resulting in high rates of avoidable medical interventions like c-sections and hysterectomies.^10^ Women typically lack the knowledge of alternative treatments required for informed decision-making. Within medicine, gynaecological issues beyond reproduction, such as perimenopause and urinary incontinence, are neglected due to funding gaps and other factors. Further, stigma and shame surrounding gynaecological issues prevent women from seeking care due to societal norms and silencing discussions around bodies. Decision-making around women's health frequently involves families and communities, complicating access to necessary care.^16^ Moreover, in some areas there is a belief that the uterus becomes a redundant, dispensable organ after childbirth.

Gaps in reproductive health services for single, LGBTIQ+, divorced and separated women are another concern. One of the panellists shared:

*“… For instance, even single working women are not routinely targeted for services by their area public health midwives. So, in Sri Lanka, each of us belongs to a designated public health midwife area, so we have access to the midwife, but they prioritize certain groups for their services. They focus on women living … with their partners or spouses … this means that a large proportion of sexually active women, including single women, LGBTIQ+ groups, divorced women, separated women, all these people who do not fall within traditional family structures, are left out.”*

## Challenges

Panellists highlighted the medicalisation of natural biological processes such as menstruation and pregnancy, which influences the way women’s health policies are spoken about, perceived and eventually treated. For instance, there is an apparent rise in hysterectomy cases that raises questions around over-medicalisation and medical practice:

*“… hysterectomy is also one of the things that is happening. We know that at some point in our life, as we start with menstruation, we could menopause, and then maybe we have some other problems. This process is natural, but in terms of hysterectomy [for such problems], is it [supply]-induced? We need to think about whether it is necessary or is it this very biomedical transitive knowledge which actually influences women’s [health]?”*

In recent years, India has seen an increase in hysterectomy operations at a young age (median age 37 years), which has been linked to a lack of viable treatment alternatives for gynaecological morbidity, health insurance, unnecessary intervention and norms around women’s bodies. In response, the Ministry of Health and Family Welfare developed guidelines to monitor unnecessary hysterectomies and promote quality care for gynaecological issues. However, there are no specific policies for post-hysterectomy and menopause management in both public and private health sectors, leaving many women deprived of necessary health care. Globally, as well as in India, early hysterectomy has been linked to increased risk of non-communicable diseases including cardiovascular disease, osteoporosis and mental health issues.^7,17^ When the age of hysterectomy is a decade before menopause, risks due to loss of oestrogen are exacerbated for a longer period. As a panellist explained:

*“… Even though we have poor health outcomes, we're very medicalized. So, we see a very paternalistic approach from healthcare providers … I mean this dynamic in which the doctor assumes the role or the midwife assumes the role of the decision maker, often overlooking the patient's preferences. We see alarmingly high rates of medical interventions, which include really high C section rates, and lots of hysterectomies performed at very young ages. Moreover, this is often driven by a combination of factors, which includes a limited understanding of alternative treatments for everyday issues … and also this idea we have of the uterus as unnecessary after childbirth. The third thing is just this lack of informed decision-making, where you don't have the information you need to make an informed decision. Due to this, there is obviously limited patient input. The doctors often will present medical options for gynaecological issues as the only viable solution without explaining potential risks and benefits. So, women are often pressured to make uninformed decisions about their health. There is obviously, again, dismissal of concerns and not highlighting what can happen if you have a radical hysterectomy and you enter early menopause in your early 30s; what does that mean for the rest of your life?”*

Another common challenge across settings is the lack of data and evidence on gynaecological and menstrual morbidities, which affects policy as well as women’s understanding of medicalisation. None of the countries collect routine population-based data on gynaecological morbidities commonly experienced by women, such as heavy menstrual bleeding.

*“… we do lack data on the burden of gynaecological and menstrual morbidities. The data which we used to collect earlier now it has been gradually stopped … Timely interventions are not there. Because of the lack of data, as well as no intervention available for the women to take care of their morbidities and gynaecological morbidities, they push these women towards not-so-good health and health experiences and even … surgical removal of the uterus.”*

In Sri Lanka and Pakistan, health data do not cover many women's health conditions and can obscure key issues such as such as health disparities based on socioeconomic status, ethnicity and other factors. For example, analysis of Demographic Health Survey (DHS)* data in Bangladesh demonstrated how women's empowerment and food security impact under-five nutrition and morbidity through pathways of health behaviour and care-seeking practices. In India, the National Family Health Survey (NFHS), building on the DHS, collects a range of data on reproductive health, intimate partner violence and basic risk factors for non-communicable disease. In response to widespread reports of early, and potentially unnecessary, hysterectomies, in 2015 the survey added specific questions to collect data on hysterectomy.

*“Interestingly, India is the only country worldwide that collects this [hysterectomy] data through the NFHS—which is a significant step and something to consider in other contexts as similar issues emerge.”*

The Longitudinal Aging Study in India (LASI) collects a wide range of information on women’s health and well-being after age 45, including mobility, mental health, chronic disease and use of services. However, both NFHS and LASI collect limited information on menstrual health and women’s experience of gynaecological morbidities.

## Areas for action

The roundtable identified several areas for action and advocacy. First, a strengthened evidence base on women’s health through the life course is imperative to influence policy and to effectively advocate for improved gynaecological health services. Data and evidence require approaches that centre women’s voices and experiences alongside population-level data collection. While the SAHELI study in India is one start, broader studies – and longitudinal studies in particular – are essential to understand women’s health over the life course in South Asia.^9^ Ethnographic studies are equally critical to gain deeper understanding of women’s bodily, emotional and societal experiences, along with the factors that affect healthcare-seeking and their choices.^18,19^ Moreover, research is needed to understand intersecting marginalities in women's health, as different groups have varying access to health care and face unique challenges.

Second, women’s health – particularly common gynaecological conditions – must be positioned within primary health care. Existing midwife-led programmes offer important lessons in how to reach women with a range of services, at the community level and through their life course. Primary health care is an ideal opportunity to address the links between gynaecological morbidity, non-communicable diseases and women’s overall health, through screening, improving preventive health literacy, primary treatment and referral as required. Self-care is important – but should adopt a holistic approach that is not limited to diagnostics, medications and technologies. Investing in continued capacity strengthening of healthcare providers and community health workers is imperative for quality service provision.

Third, involving people and communities in healthcare decisions, especially women’s participation, will be key. Broader life skills and health education in schools is a start to help change how women and others perceive and talk about their bodies. Inclusivity should ensure addressing issues related to sexual orientation and gender identity. In South Asia, as in many other settings, gender inequality and patriarchy often place the burden of diseases, medications and experiments on women. Supportive families, as well as active engagement of health workers to improve shared decision-making and respect for autonomy, will be essential to reframing women’s health choices.

Lastly, coalition-building and collaboration will be key to developing women’s health strategies specific to each context. For example, in India a broad coalition of researchers, women’s health advocates, lawyers, clinicians and professional bodies of gynaecologists, media and policymakers were key to making the rising rates of early hysterectomy a public health priority that requires a multi-level response. Expansive midwifery programmes in Sri Lanka and Pakistan offer a window of opportunity to ensure that community-based implementation realities inform policy strategies to reach women, while civil society and multi-sectoral programmes for women and girls in Bangladesh are well-positioned to raise awareness on women’s health beyond reproduction.

## Conclusion

Evidence and service delivery for women’s health need a conceptual reframing to ensure that women are “not just reproductive”. While challenges are many, promising initiatives in South Asia also illustrate how health systems can incorporate interconnected health needs in women’s lives. Addressing gynaecological health is a start to developing a life course approach to research, advocacy and services for women’s health. Ultimately, this could lead to country-level strategies that reflect a recognition of the interconnected issues that influence health and well-being through the life course, rather than in siloed life stages and organ systems.

## Footnotes

* In the time between the webinar and publication, the DHS announced it will halt its national surveys on reproductive health that covered 90 low- and middle-income countries. As noted by panellists, these surveys were a critical source of information on women’s health in South Asia.

## Provenance statement

This article was commissioned and went through internal review.
